# Observed intercamera variability in clinically relevant performance characteristics for Siemens Symbia gamma cameras

**DOI:** 10.1120/jacmp.v7i4.2376

**Published:** 2006-11-28

**Authors:** S. Cheenu Kappadath, William D. Erwin, Richard E. Wendt

**Affiliations:** ^1^ The University of Texas M.D. Anderson Cancer Center Department of Imaging Physics Houston Texas U.S.A.

**Keywords:** gamma camera, acceptance test, performance measurement, intercamera variability

## Abstract

We conducted an evaluation of the intercamera (i.e., between cameras) variability in clinically relevant performance characteristics for Symbia gamma cameras (Siemens Medical Solutions, Malvern, PA) based on measurements made using nine separate systems. The significance of the observed intercamera variability was determined by comparing it to the intracamera (i.e., within a single camera) variability. Measurements of performance characteristics were based on the standards of the National Electrical Manufacturers Association and reports 6, 9, 22, and 52 from the American Association of Physicists in Medicine. All measurements were performed using T99mc (except C57o used for extrinsic resolution) and low‐energy, high‐resolution collimation. Of the nine cameras, four have crystals 3/8 in. thick and five have crystals 5/8 in. thick. We evaluated intrinsic energy resolution, intrinsic and extrinsic spatial resolution, intrinsic integral and differential flood uniformity over the useful field‐of‐view, count rate at 20% count loss, planar sensitivity, single‐photon emission computed tomography (SPECT) resolution, and SPECT integral uniformity. The intracamera variability was estimated by repeated measurements of the performance characteristics on a single system. The significance of the observed intercamera variability was evaluated using the two‐tailed *F* distribution. The planar sensitivity of the gamma cameras tested was found be variable at the 99.8% confidence level for both the 3/8‐in. and 5/8‐in. crystal systems. The integral uniformity and energy resolution were found to be variable only for the 5/8‐in. crystal systems at the 98% and 90% confidence level, respectively. All other performance characteristics tested exhibited no significant variability between camera systems. The measured variability reported here could perhaps be used to define nominal performance values of Symbia gamma cameras for planar and SPECT imaging.

PACS numbers: 87.62.+n, 87.58.Pm, 87.58.Ce

## I. INTRODUCTION

Manufacturers of nuclear medicine gamma cameras provide system specifications based usually on the standards of the National Electrical Manufacturers Association (NEMA).^(1)^ Although the NEMA standards are quite comprehensive, a major drawback is that many of the tests described require specialized equipment and sophisticated software. These requirements make the verification of quoted NEMA system performance characteristics before clinical use somewhat difficult. Reports 6, 9, 22, and 52 from the American Association of Physicists in Medicine (AAPM) alleviate some of these difficulties by providing methods and protocols for planar and single‐photon emission computed tomography (SPECT) evaluation of gamma cameras that are both practical and time‐efficient.^(^ ^2^ ^–^ ^5^ ^)^ However, procedural differences between the NEMA and the AAPM tests undermine a direct comparison between the results using the AAPM reports and the system performance specifications claimed by the manufacturer. In addition, no published documents incorporate nominal values for the various planar acceptance tests based on the procedures described in the AAPM reports. AAPM report 52 provides nominal values for four specific SPECT acceptance test categories: rotational uniformity and sensitivity, tomographic spatial resolution, tomographic uniformity and contrast, and the accuracy of scatter correction. The reported values were based on test results from a variety of gamma cameras, but the systems tested are now somewhat outdated.

The present report gives the results of an evaluation of the intercamera (i.e., between cameras) variability of nine clinically relevant performance characteristics of Symbia gamma cameras (Siemens Medical Solutions, Malvern, PA) for both planar and SPECT imaging. The test procedures for measurement of the performance characteristics reported here were based on those defined by the NEMA standards and the AAPM reports.

## II. METHODS

Evaluation of intercamera variability was based on acceptance test measurements of nine firstgeneration Symbia gamma camera systems. These cameras were delivered brand‐new and installed in the clinic over the course of three months. Of the nine cameras, four have crystals 3/8 in. thick, and five have crystals 5/8 in. thick. The nine performance characteristics tested were intrinsic energy resolution, intrinsic and extrinsic spatial resolution, intrinsic integral and differential flood uniformity over the useful field of view, count rate at 20% count loss, planar sensitivity, SPECT resolution, and SPECT integral uniformity. All of the measurements were performed using the radionuclide T99mc (except extrinsic spatial resolution, which used C57o) and low‐energy, high‐resolution collimation when applicable. The procedures for the measurement of performance characteristics were based on those outlined in the NEMA standards and the AAPM reports, with the exception of planar spatial resolution, for which the Hander methodology was used.^(^ ^6^ ^,^ ^7^ ^)^ Table 1 lists the various planar and SPECT performance characteristics tested and the corresponding references that describe the measurement procedures.

**Table 1:** The various planar and single‐photon emission computed tomography (SPECT) performance characteristics studied and the corresponding reference that describes the measurement procedure, where FWHM is the full width at half maximum, NEMA is the National Electrical Manufacturers Association, and AAPM is the American Association of Physicists in Medicine. (a) Indicates performance characteristics tested without low‐energy, high‐resolution collimators, and (b) indicates performance characteristics not tested for intracamera variability

Performance characteristic	Units	Reference document
Planar		
Intrinsic energy resolution^(a)^	FWHM‐%	NEMA NU 1‐1994
Intrinsic integral flood uniformity^(a)^	%	NEMA NU 1‐1994
Intrinsic differential flood uniformity^(a)^	%	NEMA NU 1‐1994
Planar sensitivity	cps/μCi	NEMA NU 1‐1994
Intrinsic spatial resolution^(a,b)^	FWHM‐mm	Hander method^(^ ^6^ ^,^ ^7^ ^)^
Extrinsic spatial resolution^(b)^	FWHM‐mm	Hander method^(^ ^6^ ^,^ ^7^ ^)^
Count rate at 20% count loss	kcps	AAPM report 6
SPECT		
Spatial resolution^(b)^	FWHM‐mm	AAPM report 52
Integral uniformity	%	AAPM report 52

The observed intercamera variability was evaluated by comparing it to the observed intracamera (i.e., within a single camera) variability. The intracamera variability originates from statistical or setup variability and is assumed to be independent of the intercamera variability. To measure the intracamera variability, six of the nine performance characteristic measurements were repeated five times on a single 3/8‐in. crystal system.

The mean, standard deviation, variance, coefficient of variability (CV), and range (both positive and negative) of each performance characteristic were computed for the intercamera and intracamera variability measurements alike. Low CV values are indicative of low intercamera variability (stable performance). Because intracamera variability is usually expected to be less than or equal to intercamera variability, measurements of intracamera variability were not performed for performance characteristics with low intercamera variability—namely, planar intrinsic, planar extrinsic, and SPECT spatial resolution.

The two‐tailed *F*‐distribution was used to assess the significance of the observed intercamera variability by testing the null hypothesis that the intracamera and intercamera measurements originate from a population of measurements with the same variance.^(8)^ The *F*‐statistic depends on the measured variances of the intercamera and intracamera performance measurements. Eight and four samples (or measurements) were used, respectively, for estimating the intercamera planar performance variance (4 systems×2 detectors) and SPECT performance variance of the 3/8‐in. crystal systems. For the 5/8‐in. crystal systems, ten samples (5 systems×2 detectors) and five samples were used respectively. Five samples were used for intracamera variance estimation for both planar (one detector only) and SPECT. The *F*‐statistic, together with the sample sizes of the intracamera and intercamera variability measurements, was used to estimate the probability of rejecting the null hypothesis—that is, that the intercamera variability (or variance) was different from the intracamera variability. The probability of rejection was used as the basis for assessing whether the intercamera variability measured between the different systems was statistically significant.

## III. RESULTS

Table 2 shows the mean, standard deviation, CV, and range of each measured gamma camera performance characteristic for the two different crystal thicknesses. The planar spatial resolution (both intrinsic and extrinsic) and planar sensitivity were the only performance characteristics whose mean values were different between the 3/8‐in. and 5/8‐in. crystal systems. As expected, the thicker 5/8‐in. crystal systems demonstrated poorer spatial resolution but higher planar sensitivity. In most cases, the measured performance values were consistent with the manufacturer's specifications. The spatial resolution tests for planar intrinsic, planar extrinsic, and SPECT had the lowest CV, indicative of low intercamera variability (essentially equivalent performance) between cameras with the same crystal thickness. Because intracamera variability is expected to be less than or equal to the intercamera variability, measurements of intercamera variability for the three spatial resolution tests were not performed. On the other hand, intrinsic uniformity, count rate at 20% count loss, and SPECT integral uniformity exhibited the largest CV between systems.

**Table 2:** The mean, standard deviation, coefficient of variability (CV), and range (about the mean) of each performance characteristic measured for the 3/8‐in. and 5/8‐in. crystal gamma cameras, where UFOV is the useful field of view, LEHR is low‐energy high‐resolution collimation, and SPECT is single‐photon emission computed tomography

Performance characteristic	Crystal size (in.)	Mean value	Standard deviation	CV (%)	Range (%)
Energy resolution	3/8	9.5%	0.2%	1.9	(−4.0, 2.3)
	5/8	9.5%	0.4%	3.8	(−4.7, 6.4)
Integral uniformity UFOV	3/8	4.5%	0.4%	8.3	(−8.9, 17.7)
	5/8	4.7%	0.8%	16.9	(−21.1, 29.6)
Differential uniformity UFOV	3/8	2.6%	0.3%	10.7	(−18.2, 13.6)
	5/8	2.7%	0.3%	9.8	(−9.7, 23.3)
Intrinsic spatial resolution	3/8	3.5 mm	0.1 mm	2.2	(−2.7, 3.1)
	5/8	3.9 mm	0.1 mm	2.3	(−3.9, 3.6)
Extrinsic spatial resolution	3/8	4.4 mm	0.1 mm	2.0	(−2.3, 3.4)
	5/8	4.7 mm	0.1 mm	2.2	(−3.2, 3.7)
Count rate at 20% count loss	3/8	125.3 kcps	17.6 kcps	14.0	(−20.9, 18.9)
	5/8	129.9 kcps	13.8 kcps	10.6	(−12.8, 19.3)
Planar sensitivity LEHR	3/8	203.1 cpm/μCi	4.8 cpm/μCi	2.3	(−3.0, 2.6)
	5/8	217.0 cpm/μCi	8.8 cpm/μCi	4.1	(−7.9, 4.6)
SPECT spatial resolution	3/8	13.2 mm	0.1 mm	0.7	(−0.6, 1.0)
	5/8	13.4 mm	0.2 mm	1.2	(−1.7, 1.2)
SPECT integral uniformity	3/8	14.1%	1.5%	10.3	(−11.3, 11.7)
	5/8	13.0%	1.0%	7.6	(−9.0, 7.8)

Table 3 compares the measured variance of the intercamera and intracamera performance measurements, together with the corresponding *F*‐statistic. The calculation of the *F*‐statistic assumed that the intracamera variability was similar between the 3/8‐in. and 5/8‐in. crystal systems. The null hypothesis was rejected for planar sensitivity by the *F*‐statistic at the 99.8% confidence level for both the 3/8‐in. and 5/8‐in. crystal systems. The null hypothesis was rejected for intrinsic integral uniformity by the *F*‐statistic (at the 98% confidence level) only for the 5/8‐in. crystal systems. The null hypothesis was marginally rejected by the *F*‐statistic (at the 90% confidence level) for energy resolution for the 5/8‐in. crystal systems. The intercamera variability was found to be consistent with the intracamera variability for all other performance characteristics measured.

**Table 3:** The variance and sample size of the intercamera and intracamera performance measurements, together with the corresponding F‐statistic for the 3/8‐in. and 5/8‐in. crystal gamma camera systems. The F‐statistic values indicated by (a), (b), and (c) correspond to rejection of the null hypothesis at the 90%, 98%, and 99.8% confidence level respectively

	Variance (number of samples)			*F*‐statistic	
Performance characteristic	Intra 3/8‐in.	Inter 3/8‐in.	Inter 5/8‐in.	3/8‐in.	5/8‐in.
Energy resolution	0.017 (5)	0.034 (8)	0.128 (10)	2.0	7.4^(a)^
Integral uniformity	0.04 (5)	0.14 (8)	0.62 (10)	4.0	17^(b)^
Differential uniformity	0.06 (5)	0.08 (8)	0.07 (10)	1.3	1.2
Count rate at 20% count loss	63 (5)	309 (8)	190 (10)	4.9	3.0
Planar sensitivity	0.4 (5)	22.6 (8)	77.7 (10)	54^(c)^	186^(c)^
SPECT integral uniformity	3.8 (5)	2.1 (4)	1.0 (5)	1.8	3.9

**Figure 1:**
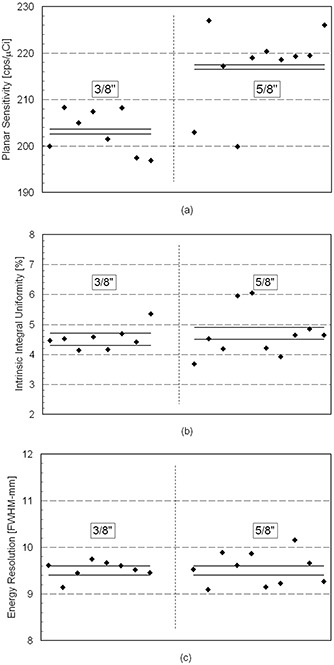
Measurements (filled diamonds) of (a) planar sensitivity, (b) intrinsic integral uniformity, and (c) intrinsic energy resolution, where the abscissas correspond to the individual detectors (two per camera) of the nine gamma cameras tested. Cameras were separated into two groups corresponding to the gamma camera crystal thickness (3/8 in. and 5/8 in.). The upper (lower) horizontal lines in each plot correspond to the mean performance value plus (minus) 1‐σ of the measured intracamera variability for the two crystal thicknesses. The significance of the intercamera performance variability roughly corresponds to the number and magnitude of deviation of the measurements that fall outside the 1‐σ range indicated

Fig. 1 shows the measured values of planar sensitivity, intrinsic integral uniformity, and intrinsic energy resolution of the individual detectors (two per camera) for the nine gamma cameras tested. The measurements in each plot are separated along the abscissa into two groups (3/8 in. and 5/8 in.) corresponding to the crystal thicknesses of the gamma cameras. It is important to note that the variability results are independent of the exact placement of the detector measurements along the abscissa within each group of crystal thicknesses. The significance of the intercamera performance variability (computed using the *F*‐statistic) correspond to the number of measurements that fall outside the 1‐σ range (Fig. 1), as well as to the magnitude of the deviations. Consistent with the estimated significance of the intercamera variability (*F*‐statistic), most (17 of 18) sensitivity measurements fall outside the 1‐σ range for both crystal systems, and numerous (6 of 10) intrinsic integral uniformity and (8 of 10) intrinsic energy resolution measurements fall outside the 1‐σ range for the 5/8‐in. crystal system.

## IV. DISCUSSION

Planar sensitivity calculations require accurate measurement of the source activity and careful experimental setup to minimize self‐absorption by the source. Errors in the measurement of source activity, varying amounts of self‐absorption in various source preparations, and differences in collimator efficiency between cameras may explain some of the observed intercamera variability in the planar sensitivity measurements. Although the cameras were calibrated and tuned before testing began, drifts in the photomultiplier tube gain and high‐voltage settings may explain some of the observed intercamera variability in the intrinsic integral uniformity and intrinsic energy resolution measurements.

With the exception of spatial resolution—and, to a lower degree, the count rate performance and the planar sensitivity—the performance characteristics do not change markedly between the 3/8‐in. and the 5/8‐in. crystal systems, suggesting that the statistical properties of the individual performance measurements are not widely different between the two camera systems. The only difference between the 3/8‐in. and the 5/8‐in. crystal systems are their nominal crystal thickness, given that both gamma camera systems use identical detector hardware and readout electronics. The aforementioned observations, together with the fact that the intracamera variability is believed to originate from the statistical uncertainty and the setup variability of each measurement, lend strong support to the assumption that the intracamera variability measurements are independent of the crystal thickness in the gamma cameras.

## V. CONCLUSIONS

Most of the planar and SPECT performance characteristics tested (except planar sensitivity, intrinsic integral uniformity, and intrinsic energy resolution) exhibited no significant variability between the various camera systems. The planar sensitivity was found to be the most variable performance characteristic at a high significance (>99% confidence level), for the 3/8‐in. and 5/8‐in. crystal systems alike. Integral uniformity was found to be the second most variable performance characteristic (>98% confidence level), but only for the 5/8‐in. crystal systems. The intercamera variability of energy resolution was found be only marginally significant (>90% confidence level), but again, only for the 5/8‐in. crystal systems.

This report evaluates nine different performance characteristics for both planar and SPECT acceptance testing, but its scope is limited to Siemens Symbia gamma cameras. Nevertheless, the measured intercamera and intracamera variability reported here may be used to define acceptable performance limits for Siemens Symbia gamma cameras (perhaps as a class standard)—at least for the performance characteristics for which no statistically significant intercamera variability was observed. One possible method of stipulating the limit of acceptability could be to use the mean value of the measured performance characteristic minus or plus two standard deviations (2‐σ), depending on whether a lower or an upper limit is to be specified. Table 4 enumerates the acceptable performance values based on the above criteria and the measured intercamera variability.

**Table 4:** The proposed acceptable performance values (perhaps specified as a class standard) stipulated as the mean performance value plus or minus 2‐σ of the measured intercamera variability (depending on whether an upper or a lower limit), for Siemens Symbia gamma cameras with 3/8‐in. and 5/8‐in. crystals, where FWHM is full width at half maximum, and SPECT is single‐photon emission computed tomography

		Crystal thickness	
Performance characteristic	Units	3/8‐in.	5/8‐in.
Planar			
Intrinsic energy resolution	FWHM‐%	<9.9	<10.3
Intrinsic integral flood uniformity	%	<5.3	<6.3
Intrinsic differential flood uniformity	%	<3.2	<3.3
Intrinsic spatial resolution	mm	<3.7	<4.1
Extrinsic spatial resolution	mm	<4.6	<4.9
Planar sensitivity	cps/μCi	>193	>200
Count rate at 20% count loss	kcps	>90	>102
SPECT			
Spatial resolution	FWHM‐mm	<13.4	<13.8
Integral uniformity	%	<17	<15
